# Application of the Cobas 4800 System for the Detection of High-Risk Human Papillomavirus in 5650 Asymptomatic Women

**DOI:** 10.1155/2020/1635324

**Published:** 2020-03-21

**Authors:** Shanyun Wang, Xijun He, Fansu Meng, Qianyi Pan, Lin Zhang, Jianfeng Zeng

**Affiliations:** ^1^Department of Preventive Healthcare, Zhongshan Hospital of Traditional Chinese Medicine, 3 Kangxin Road, Western District, Zhongshan City, 528400 Guangdong Province, China; ^2^Department of Critical Care Medicine, Zhongshan Hospital of Traditional Chinese Medicine, 3 Kangxin Road, Western District, Zhongshan City, 528400 Guangdong Province, China; ^3^Department of Surgery (1st Division), Zhongshan Hospital of Traditional Chinese Medicine, 3 Kangxin Road, Western District, Zhongshan City, 528400 Guangdong Province, China

## Abstract

High-risk papillomavirus (HR-HPV) testing combined with cytology improves the detection of cervical lesions and increases length of screening intervals. For a population-based HR-HPV survey, testing automation is in great need. The Cobas 4800 HPV Test System is a fully automated assay that can simultaneously detect HPV16, HPV18, and other 12 pooled HR-HPV genotypes. This system has been employed for HR-HPV screening in a number of countries; however, such application in a large population in China has not been documented. In this study, we employed the Cobas 4800 HPV Test System to detect HR-HPV in cervical cytology specimens collected from a total of 5650 asymptomatic women from a region of South China. We reported the following: (1) the prevalence of the 14 genotypes of HR-HPV was 12.96%; (2) for those with HR-HPV infection, 2.25% were positive for HPV16, 0.50% for HPV18, 9.15% for pooled 12 HPV types, and 1.06% for multiple HPV infection; and (3) there was no significant difference in the HR-HPV prevalence among different age groups. HPV16 and HPV18 have been shown to be the predominant HPV types found in cervical cancer patients in some regions in China, indicating that a fully automated assay like the Cobas 4800 HPV Test System is especially valuable for population-based HR-HPV screening in these regions as this assay can concurrently detect HPV16 and HPV18.

## 1. Introduction

Human papillomavirus (HPV) is a double-stranded DNA virus of the Papillomaviridae family [1]. There are more than 200 genotypes of HPV that have been identified, among which 15 (16, 18, 31, 33, 35, 39, 45, 51, 52, 56, 58, 59, 68, 73, and 82) are highly associated with cervical cancer [2, 3]. Cervical cancer is the fourth most common cancer in women worldwide [4] and the sixth most common cancer in women in China [5]. To reduce the burden of cervical cancer, early identification of cervical lesions that likely progress to invasive cancer is of paramount importance. Cervical cytology plays an important role in the prevention of cervical cancer [6]. The addition of HPV testing to cytology results in an increased detection of high-grade cervical lesions, and it is therefore recommended that screening of high-risk HPV (HR-HPV) be incorporated into cytology to improve disease detection and increase length of screening intervals [7, 8].

To date, various methods have been reported for HPV genotyping and detection [9, 10]. For population-based screening, testing automation is especially useful as automation possesses the ability to process a large number of samples in a short time and reduce human errors that can occur in manual operations. The Cobas 4800 HPV Test System (Roche Molecular Systems Inc., Rotkreuz, Switzerland), fully automated, is designed to simultaneously detect a total of 14 HR-HPV types: HPV16 individually, HPV18 individually, and 12 pooled HR-HPV genotypes (31, 33, 35, 39, 45, 51, 52, 56, 58, 59, 66, and 68) [10]. This system has been approved by the U.S. Food and Drug Administration for HR-HPV screening. The Cobas 4800 HPV Test System consists of 2 parts, i.e., a Cobas x 480 Instrument that is used for automation of nucleic acid purification and PCR sample pipetting and a Cobas z 480 Analyzer that is for automatic real-time PCR amplification and subsequent type-specific hybridization. Although the use of the Cobas 4800 System for HR-HPV testing has been reported in a number of countries [11–16], such application in a large population in China has not been documented. In this study, we employed the Cobas 4800 HPV Test System to detect HR-HPV in cervical cytology specimens collected from a total of 5650 asymptomatic women.

## 2. Methods

This study was approved by the Medical Research Review Committee of Zhongshan Hospital of Traditional Chinese Medicine. Informed consent was obtained from all participants.

### 2.1. Cervical Sample Collection

Asymptomatic women aged 20-65 years were enrolled from January 2018 to December 2018 for this study with the testing cost covered by study subjects or their employers or in combination. The inclusion criteria include being 20 years or older, being not pregnant, having intact uterus, being sexually active, or having history of sexual activity. All subjects were informed of not using vaginal medications, vaginal contraceptives, or lubricants for 48 hours before cervical sampling. Cervical cytology specimens were collected using the Cervex-Brush Combi device (Rovers Medical Devices) as follows: the endocervical sampling part of the device was inserted into the endocervical canal to allow the lateral bristles to fully contact the ectocervix, and the device was then rotated two full turns clockwise. After withdrawal, the device with the harvested cells was immediately inserted into a vial containing PreservCyt Solution (Hologic Inc., Bedford, MA, USA) and vigorously swirled. Samples were used for HPV DNA detection as described below.

### 2.2. HR-HPV Detection Using the Cobas 4800 HPV Test System

Lysis Buffer, Wash Buffer, Elution Buffer, HPV Positive and Negative Control Kits, and all other reagents were purchased from Roche Molecular Systems Inc. Each sample had an internal control, *β*-globin, to monitor cell adequacy, and each run included a set of HPV Positive and Negative Controls. DNA extraction and purification were done with the Cobas x 480 Instrument according to the manufacturer's instructions. Briefly, the PreservCyt Solution samples were vortexed and placed on the sample carrier, and the reagents, e.g., Lysis Buffer, Wash Buffer, and Elution Buffer, were loaded in respective reagent reservoir carriers. After sample and reagent loading, DNA preparation was completed automatically and final DNA products were collected into a microwell plate. Subsequently, the microwell plate containing DNA was manually sealed and loaded on the Cobas z 480 Analyzer, and the amplification and hybridization were completed automatically.

### 2.3. Statistical Analysis

Statistical analysis was performed using SPSS 13.0 (IBM Corp., Armonk, NY, USA). The *χ*^2^ test was used to assess categorical variables. All statistical tests were 2-sided, and *P* values less than 0.05 were considered statistically significant.

## 3. Results

A total of 5650 asymptomatic women aged 20-65 years with a mean age of 38.8 ± 12.2 years met the study requirement and were included in this study. Of these participants, 732 (12.96%) were detected as HR-HPV-positive, including 127 (2.25%) with HPV16 infection, 28 (0.50%) with HPV18, 517 (9.15%) with pooled 12 HPV types, and 60 (1.06%) with multiple HPV infection (HPV16 mixed with HPV18, HPV16 mixed with 12 pooled types, HPV18 mixed with 12 pooled types, or HPV16 mixed with HPV18 and 12 pooled types) (Table 1).

According to the age, participants were divided into 4 groups: 20-30, 31-40, 41-50, and >51 years. As shown in Table 2, the 31-40 years group had the highest HR-HPV infection rate of 15.32% followed by the groups of >50 years, 41-50 years, and 20-30 years, although no statistical significance was observed among the infection rates of these groups.

## 4. Discussion

In this study, we used the Cobas 4800 System to detect HR-HPV in 5650 asymptomatic women and found the following: (1) the prevalence of the 14 genotypes of HR-HPV was 12.96%; (2) of the 732 HR-HPV-positive women, 2.25% were positive for HPV16, 0.50% for HPV18, 9.15% for pooled 12 HPV types, and 1.06% for multiple HPV infection; and (3) there was no significant difference in the HR-HPV prevalence among different age groups.

Both the RealTime High Risk HPV Test from Abbott Molecular Inc. (Des Plaines, IL, USA) and the Cobas 4800 HPV Testing System are clinically validated, fully automated PCR-based assays for the detection of HR-HPV [10]. One advantage of these 2 assays is that they can concurrently detect and distinguish HPV16 and HPV18, which is important for cervical cancer screening as HPV16 and HPV18 account for approximately 70% of cervical cancers globally [17, 18]. Few studies have compared these 2 assays in detecting HR-HPV in clinical samples. Park et al. tested 365 cervical swab specimens and reported that the RealTime High Risk HPV Test presented a sensitivity of 78.3% with a specificity of 99.2% while the sensitivity and specificity of the Cobas 4800 System were 91.7% and 97.0%, respectively [19]. Cuzick et al. screened 6000 women and revealed that 16.0% were detected to be HR-HPV-positive by the Cobas 4800 System while 13.4% were detected to be HR-HPV-positive by the RealTime High Risk HPV Test [20]. These data indicate that the Cobas 4800 System possesses greater detection sensitivity.

Several clinically validated molecular systems for the detection of HR-HPV DNA have been included by the World Health Organization in 2016 in the manual *Integrating HPV Testing in Cervical Cancer Screening Programs-a Manual for Program Managers* (https://www.paho.org/hq/dmdocuments/2016/manual-VPH-English---FINAL-version.pdf). These tests consist of hybridization-based assays, i.e., the Hybrid Capture 2 technique and the CareHPV test, and PCR-based assays, e.g., the Cobas HPV test, the Abbott RealTime High Risk HPV test, the Cervista HPV HR test, and the BD HPV assay. All PCR-based assays require DNA isolation while hybridization-based assays do not. Of all the methods, the Cervista HPV HR Test takes 6-7 hours and the Abbott RealTime High Risk HPV assay takes 6-8 hours to run, which may not fulfill the goal of “see and treat” the same day. In contrast, all the rest methods including the Cobas 4800 System, however, require a maximal run time of approximately 5 hours, suitable for patients to complete “see and treatment” in one visit.

Globally, HR-HPV prevalence varies from continent to continent [21]. In China, for the survey of HR-HPV prevalence, different methods have been used [22–25]. Wu et al. employed the Hybrid Capture 2 Assay (Qiagen, Hilden, Germany) for the detection of HR-HPV of 13 pooled types (HPV16, 18, 31, 33, 35, 39, 45, 51, 52, 56, 58, 59, and 68) in a total of 4215 women aged 17-54 years from 5 different geographical areas of China and discovered that HR-HPV prevalence was 12.4% [22]. Using the same method as Wu et al. [22], Zhao et al. found that 15.2% of 1274 women from a region in North China were positive for HR-HPV [23]. Chen et al. employed a HPV Genotyping Kit (Yaneng Biotechnology Limited Corp., Shenzhen, China) for a total of 961,029 HPV tests for women aged 16 to 83 years from Southeastern China and revealed a HR-HPV infection rate of 16.61% [24]. Jing et al. applied a mass spectrometric assay for HPV-DNA testing in 78,355 women aged 18 to 75 years from South China and reported a <7.3% HR-HPV prevalence rate [25]. These studies demonstrate a HR-HPV infection rate ranging approximately 10-15% in Chinese women, which is in agreement with our finding. However, the method we used in the present study is simpler and less laborious compared with those used in previous investigations [22–25].

Two studies with the largest sample size conducted in China have compared HR-HPV prevalence rates among age groups. Zeng et al. retrospectively analyzed 671,163 HPV tests performed using the Hybrid Capture 2 method from 2007 to 2014 and demonstrated the highest HR-HPV prevalence of 36.9% in the age group of <20 years while the HR-HPV-positive rates for the age groups of 20-30, 30-40, and 40-50 years were 24.4%, 19.7%, and 19.9%, respectively [26]. HR-HPV testing with the largest sample size (961,029 samples) carried out by Chen et al. again shows the highest prevalence rate of HR-HPV in the age group of <20 years (22.3%) while the prevalence rates for the age groups of 20-30, 30-40, and 40-50 years are 16.8%, 15.2%, and 15.9%, respectively [24]. These studies reveal a slightly higher prevalence rate in the group of 20-30 years than in the groups of 30-40 and 40-50 years, which is in contrast with our results, as we observed the lowest HR-HPV prevalence in the group of 20-30 years. This discrepancy may occur due to the following factors: (1) younger women nowadays have more access to health education programs, especially online programs using electronic devices, and (2) younger women are more eager to learn and accept new knowledge on sanitation and sexual health.

HPV16 is the most common type of HR-HPV found in cervical samples of women from North (Beijing and Henan, Shanxi, and Xinjiang Provinces), Southeast (Shanghai and Zhejiang Province), and South China (Guangdong Province) regions, with a positive rate ranging from 1.5 to 3.6% [22–25], which is in line with a 2.25% HPV16 infection rate observed in our study subjects who are from South China. HPV16 is responsible for approximately 50% of cervical cancer globally [17, 18]. While the information on HR-HPV genotype distribution in Chinese cervical cancer patients is still limited, Li et al. reported that 1818 out of 2309 cervical cancer patients (78.7%) from West China were positive for HPV16, and approximately 90% of these patients were positive for HPV16 and HPV18 [27]. A 53.2% infection rate for HPV16 and HPV18 was also shown in 326 cervical cancer patients from Fujian Province in Southeast China [28]. These data indicate that HPV16 and HPV18 are the predominant HPV types found in cervical cancer patients in these regions. A fully automated assay like the Cobas 4800 HPV Test System that can simultaneously detect and differentiate HPV16 and HPV18 will be especially valuable for population-based HR-HPV screening in regions that have a HPV16 and HPV18 infection dominance.

## Figures and Tables

**Table 1:** Positive rates of different types of HR-HPV in 5650 asymptomatic women.

HPV type	Positive, *N* (%)
HPV16	127 (2.25%)
HPV18	28 (0.50%)
Pooled 12 HPV types	517 (9.15%)
Multiple	60 (1.06%)

Multiple HPV infections include HPV16 mixed with HPV18, HPV16 mixed with 12 pooled types, HPV18 mixed with 12 pooled types, or HPV16 mixed with HPV18 and 12 pooled types.

**Table 2:** HR-HPV infection rates in different age groups.

Group (years)	Total participants	HPV16, *N* (%)	HPV18, *N* (%)	12 pooled HPV, *N* (%)	Multiple HPV, *N* (%)	Total, *N* (%)
20-30	1201	16 (1.33%)	7 (0.58%)	93 (7.74%)	10 (0.83%)	126 (10.49%)
31-40	2036	67 (3.29%)	12 (0.59%)	208 (10.22%)	25 (1.23%)	312 (15.32%)
41-50	1825	29 (1.59%)	6 (0.33%)	164 (8.99%)	20 (1.10%)	219 (12.00%)
>50	588	15 (2.55%)	3 (0.51%)	52 (8.84%)	5 (0.85%)	75 (12.76%)
Total	5650	127 (2.25%)	28 (0.50%)	517 (9.15%)	60 (1.06%)	732 (12.96%)

## Data Availability

The data used to support the findings of this study are available from the corresponding author upon request.
